# QT dispersion increases with low glomerular filtration rate in patients with coronary artery disease

**Published:** 2014

**Authors:** Murat Celik, UygarCagdas Yuksel, Yalcin Gokoglan, Baris Bugan, Emre Yalcinkaya, HilmiUmut Unal, Turgay Celik, Atila Iyisoy, Selim Kilic

**Affiliations:** 1 Murat Celik, Department of Cardiology, Gulhane Military Medical Academy, School of Medicine, Ankara, Turkey.; 2 UygarCagdasYuksel, Department of Cardiology, Gulhane Military Medical Academy, School of Medicine, Ankara, Turkey.; 3 YalcinGokoglan, Department of Cardiology, Gulhane Military Medical Academy, School of Medicine, Ankara, Turkey.; 4 BarisBugan, Department of Cardiology, Gulhane Military Medical Academy, School of Medicine, Ankara, Turkey.; 5 EmreYalcinkaya, Department of Cardiology, Gulhane Military Medical Academy, School of Medicine, Ankara, Turkey.; 6 Hilmi Umut Unal, Department of Nephrology, Gulhane Military Medical Academy, School of Medicine, Ankara, Turkey.; 7 TurgayCelik, Department of Cardiology, Gulhane Military Medical Academy, School of Medicine, Ankara, Turkey.; 8 AtilaIyisoy, Department of Cardiology, Gulhane Military Medical Academy, School of Medicine, Ankara, Turkey.; 9 SelimKilic, Department of Epidemiology, Gulhane Military Medical Academy, School of Medicine, Ankara, Turkey.

**Keywords:** Coronary artery disease, Glomerular filtration rate, QT dispersion

## Abstract

***Objective:*** We aimed to evaluate the relationship between estimated glomerular filtration rate (eGFR) and QT dispersion (QTd) in patients with coronary artery disease (CAD).

***Methods:*** Sixty patients(mean age 62.72 ± 12.48 years) included 46 male, (mean age 60.89 ± 12.70 years)and 14 female (mean age 68.71± 9.86 years) were enrolled in this study. Patients were divided into 2 groups according to their eGFR using the 6 variable MDRD equation. Group 1 consisted of patients with estimated eGFR<60 ml/min/1.73m^2^ and Group 2 consisted of patients witheGFR ≥ 60 ml/min/1.73m^2^.

***Results:*** Baseline patient characteristics were homogeneous in both groups except for age, gender and smoking.Also, the extent of CAD was similar in both groups (p > 0.05) QTd values were found higher in group 1 than those of group 2 (57.23 ± 40.65 ms vs. 31.23 ± 14.47 ms, p = 0.002). After adjustment for age, gender and smoking using one-way ANCOVA test, statistically significant difference in QTd still existedbetween the groups (p=0.038).

***Conclusion:***QTd tends to be higher in patients with poor renal function independent of severity of angiographical CAD. QTd may be a potentially useful non-invasive test in the management of patients with poor renal function, especially those with CAD.

## INTRODUCTION

Impairment of renal function is associated with increased cardiovascular risk.^1^ Cardiovascular diseases, such as coronary artery disease (CAD) and heart failure are the leading cause of death in patients with renal insufficiency.^2^ There is a strong correlation between the degree of renal dysfunction and the presence of CAD. However, the role of parameters showing renal function on predicting the extent and severity of CAD is still conflicting. ^3^ Deterioriation of renal function is associated with a number of predisposing risk factors for cardiovascular disease such as increased neurohormonal activation, inflammation, increased oxidant stres, insulin resistance, increased plasma asymmetric dimethylarginine (ADMA) level, hyperhomocysteinemia,higher serum lipoprotein (a) levels and increased prothrombotic factors. ^4^ However, it is not yet well understood that whether impairment of renal function increases the cardiovascular risk independently or together with associated risk factors.

QT dispersion (QTd)is defined as the difference between the longest and shortest QT interval recorded from surface electrocardiogram (ECG) and is an indirect, non-invasiveparameter of ventricular repolarisation and homogeneity. The prognostic value of QTd has been investigated in a number of cardiovascular diseases and conditions, such as hypertension, angina pectoris, paroxysmal atrial fibrillation and coronary artery bypass graft (CABG) operation. QTd is a well-established predictor of morbidity and mortality with an ever-growing use in clinical practice.

In previous studies ^5^^-^^7^, it was shown that QTd increased significantly in patients with chronic end-stage renal failure, particularly in patients receiving hemodialysis, which increases QTd after eachsession. ^7^ However, there is limited data in literature investigating the relationship between estimated glomerular filtration rate (eGFR) and QTd in patients with mild to moderate renal insufficiency and/or chronic renal failure not receiving hemodialysis. In this study, we aimed to investigate the role of eGFR on QTd in patients with CAD.

## METHODS

***Patients: ***Patients in whom coronary angiography (CAG) was performed due to a high clinical suspicion of CAD were included in the study. Patients with CAD were evaluated. Acute myocardial infarction, in the last one month, unstable angina pectoris, type 1 diabetes mellitus, uncontrolled hypertension (systolic blood pressure > 190 mm Hg), atrial fibrillation, bundle branch block, presence of permanent pacemaker or automatic implantable cardioverter device, use of antiarrhythmic drugs that may affect the QT interval, previous CABG operation, severe systemic inflammatory disease, hepatic failure and receiving hemodialysis were regarded as exclusion criteria.Sixty patients met the inclusion criteria and constituted the study population. On admission, arterial blood pressure and heart rate measurement, resting 12-lead electrocardiography (ECG), transthoracic echocardiography, whole blood count and standard biochemical tests were performed in all patients. CAG was performed in all patients. To prevent the contrast-induced nephropathy, periprocedural IV hydration with normal saline was started and administration of oral NAC at 600 mg twice daily on the day before and the day of the procedure was given in patients with reduced eGFR. Also, attention was paid to the use of low-osmolality contrast media in these patients.Patients were divided into two groups according to their eGFR values measured using the six variable modifications of diet in renal disease (MDRD) equation. Group-1 consisted 30 patients with CAD and eGFR< 60 mL/min/1.73m^2^ and Group-2 was consisted 30 patients with CAD and eGFR> 60 mL/min/1.73m^2^.The regional ethics committee approved the study protocol. 

***Surface electrocardiography and QT analyses: ***A resting 12-lead surface ECG with a paper speed of 50 mm/s and a signal size of 10 mm/mV (Hewlett Packard M1700A; Hewlett Packard, Houston, TX) was recorded before CAG in the morning period in order to prevent circadian variation. Then, all ECGs were scanned using a high-resolution scanner (HP Deskjet F2480 Allin-One Printer, 4800 × 1200 dpi; Hewlett Packard) and transferred to the computer. The measurements were performed on screen using digital magnification with a high-performance graphic program (Adobe Photoshop CS2; Adobe, San Jose, CA). The ECGs were zoomed in times and ECG gridlines were assessed appropriately to provide correct conversion to milliseconds and to confirm that the image was perpendicular to the measuring tool. QT interval was measured from the onset of QRS complex to the end of T wave defined by the return of the T wave to the isoelectric TP baseline and averaged in every lead. When T wave was negative, the point where the T wave returned to the isoelectric TP baseline was taken as the end of T wave. When U wave was present, the nadir of the curve between T and U wave was taken as the end of T wave. If the end of T wave was not determined properly in any lead, then this particular lead was excluded from analysis. After these analyses, the difference between the maximum and minimum QT interval was defined as QTd (QTd (ms) = QTmaximum - QTminimum). All measurements were performed by two separate investigators blinded to each other’s results and patients’ clinical data.

***Statistical analysis: ***After all error controls and corrections, statistical analyses and calculations were performed using the SPSS 16.0 Statistical Package Program for Windows (SPSS, Chicago, IL). We used one-sample Kolmogorov–Smirnov and Levene tests to determine the distribution characteristics of variables and homogeneity of variance. All results for continuous variables with normal distribution were expressed as mean ± standard deviation (SD), skewed variables as median (interquartile range — IQR) and categorical variables as percentages. Independent samples t-test was used to compare the continuous variables with normal distribution, Mann-Whitney U-test was used for skewed variables between two groups, andχ2 test and χ2 likelihood ratio were used for categorical variables. Analysis of covariance (ANCOVA) was used to analyze the confounding effects of variables on QTd values. The variables for ANCOVA were age, gender and smoking. Intra- and inter observer variabilities were calculated in terms of relative error. Differences were considered significant at p< 0.05.

## RESULTS

A total of 60 patients (46 male) aged 35–88 years (mean age 62.72 ± 12.48 years) 14 female (mean age 68.71± 9.86 years) were enrolled in this study. The mean age of patients was 69.3 ± 12.06 years in group 1 and 56.13 ± 9.03 years in group 2 (p < 0.001). The baseline socio demographic and clinical characteristics of the study population are presented in Table-I. The two groups were similar with regard to systolic blood pressure, diastolic blood pressure, echo cardiographic findings and risk factors (smoking, HT, HL, DM, previous CAD). However, there were statistically significant differences for age, gender and smoking between the groups. Coronary angiography findings of patients included in the study are presented in Table-II. Number of diseased vessel and number of totally occluded vessel did not differ significantly between the groups (p=0.355 and p=0.301, respectively).

**Fig.1:**
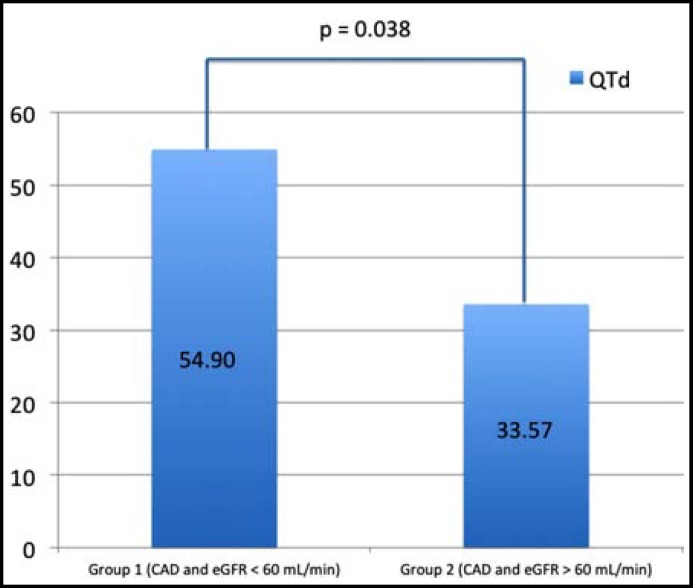
QTd values after adjustment for age, sex and smoking using one-way ANCOVA (Analysis of covariance) test

**Table-I:** Baseline socio demographic and clinical characteristics

	*Group 1 (n=30)*	*Group 2 (n=30)*	*p*
Age (years)	69.3 ± 12.06	56.13 ± 9.03	< 0.001
Male n(%)	19(%63)	27(%90)	0.037
Systolic blood pressure [mmHg]	132.30 ± 23.81	126.80 ± 20.65	0.343
Diastolic blood pressure [mmHg]	72.23 ± 12.45	73.43 ± 13.96	0.727
Left ventricular internal dimension-diastole (mm)	52.63 ± 8.22	51.73 ± 6.48	0.640
Left ventricular ejection fraction (%)	44.83 ± 13.23	49.15 ± 12.66	0.202
Glomerular filtration rate (mL/min)	36.62 ± 16.63	84.66 ± 16.65	<0.001
Risk factors			
Smoking, n(%)	9 (%30)	17 (%56)	0.037
Previous CAD, n(%)	18 (%60)	19 (%63)	0.791
HT, n(%)	21 (%70)	16 (%53)	0.184
DM, n(%)	10 (%33)	12 (%40)	0.592
HL, n(%)	6 (%20)	6 (%30)	0.371

Values are expressed as mean values ± s.d. or number (percentage).

CAD: Coronary artery disease, HT: Hypertension, DM: Diabetes mellitus, HL: Hyperlipidemia.

**Table-II:** Coronary angiography findings of patients

		*Group1 (n=30)*	*Group2 (n=30)*	*p*
Number of diseased vessels, n(%)	0	0	0	0.355
	1	0	1 (%3)	
	2	**0**	1 (%3)	
	3	30 (%100)	28 (%94)	
Number of totally occluded vessels, n(%)	0	3 (%10)	2 (%6)	0.301
	1	12 (%40)	18 (%60)	
	2	15 (%50)	10 (%34)	
	3	0	0	

Values are expressed as numbers (percentage)

**Table-III:** Differences in QT interval measurements between two groups according to GFR

	*Group 1 (n=30)*	*Group 2 (n=30)*	*p*
Heart rate (bpm)	77.53 ± 16.37	73.20 ± 12.23	0.250
QTmax (ms)	456.97 ± 53.66	426.97 ± 43.45	0.084
QTmin(ms)	400 ± 52.47	396.67 ± 42.86	0.293
QTd	57.23 ± 40.65	31.23 ± 14.47	0.002

Values are expressed as mean values ± s.d.

Patients with low eGFR (< 60 mL/min/1.73m^2^) and CAD had higher QTd values compared with those of normal eGFR (>60 mL/min/1.73m^2^) and CAD (57.23 ± 40.65 ms vs. 31.23 ± 14.47 ms, p value 0.002). However, QTmax and QTmin values did not differ significantly between the groups (for QTmaximum 456.97 ± 53.66 ms vs. 426.97 ± 43.45 ms, p value 0.084 and for QTminimum 400 ± 52.47 ms vs. 396.67 ± 42.86 ms, p value 0.293) (Table-III). After adjustment for age, sex and smoking using one-way ANCOVA (Analysis of covariance) test, statistically significant difference for QTd still existed between the groups. After adjustment, QTd value for group 1 was 54.90±35.59 and QTd value for group 2 was 33.57^.^35.59 (p = 0.038) (Fig.1).

## DISCUSSION

In this study, significant relationship between QTd and eGFR was observed in patients with CAD. We found that, QTd was significantly higher in patients with low eGFR than those of normal eGFR and CAD.

Patients with renal failure are more predisposed to cardiovascular disease compared to patients with normal kidney function. ^8^ In HDFP (the Hypertension Detection and Follow-up Program) study, it was shown that cardiovascular mortality increased depending on the elevations of serum creatinine level. ^9^ This observed increase in mortality rate was primarily related to level and duration of renal failure, however, can also occur in early stage of the disease.

eGFR is the most appropriate and simple way of assessing renal function. Besides, Aras et al. ^10^ showed that eGFR is a useful diagnostic parameter for predicting presence of CAD in women. AneGFR level of ≤ 60 ml/min/1.73m^2^ represents moderately renal failure. Most of the prospective cohort studies demonstrated an inverse relationship between the degree of renal dysfunction and cardiovascular morbidity and mortality, even in patients with moderate renal failure. ^11^ This inverse correlation was more evident in patients with eGFR< 60 ml/min/1.73m^2^when compared with patients with GFR> 60 ml/min/1.73m^2^. ^11^ In addition, moderately decreased eGFR was accepted as major cardiovascular risk factor and all patients with moderately decreased eGFR were considered as high risk according to JNC VII report (Joint National Committee on Prevention, Detection, Evaluation, and Treatment of High Blood Pressure). ^12^ Therefore, eGFR of 60 ml/min/1.73m^2^ was used as a threshold limit value in our study. 

Description of the abnormalities of cardiac repolarization and myocardial homogeneity from the surface ECG has long been the focus of attention. QTd has been shown as a non-invasive, cheap, simple and significant clinical index indicating repolarization heterogeneity of ventricular myocardium. QTd has been widely investigated, and found as a risk factor for severe malignant ventricular arrhythmias and sudden cardiac death in a number of cardiac (long QT syndrome, acute myocardial infarction, heart failure, left ventricular hypertrophy, idiopathic dilated cardiomyopathy, hypertrophic cardiomyopathy, aortic stenosis, etc.) and non-cardiac (diabetes mellitus, rheumatoid arthritis, ankylosing spondylitis, anorexia nervosa, electrolyte imbalance, severe burns, etc.) disease and conditions. ^13^^-^^18^ Cardiovascular mortality was found to be increased by 34% for each 17 ms increase in QTd in multivariate analyzes. ^19^ Patients with chronic renal failure had a greater QT interval and QTd compared with the control subjects ^6^, and it is shown that increase in QTd was more pronounced in patients with chronic end-stage renal disease receiving renal replacement therapy such as hemodialysis or periton dialysis. ^5^^-^^7^ However, there is no clinical study analyzing the impact of early stages of chronic renal failure on QTd. In this study, we found a statistically significant QTd prolongation in CAD patients with low eGFR< 60 ml/min/1.73m^2^ compared with those of CAD and eGFR ≥ 60 ml/min/1.73m^2^.

Deterioration of renal function has a negative effect on cardiovascular biology and physiology and can lead to deterioration of ventricular repolarization and myocardial homogeneity. In patients with renal failure, QTd prolongation may occur due to several mechanisms. Ventricular dilation, myocardial fibrosis (especially parathormone-dependent intermyocardiocytic fibrosis), myocardial calcification, myocyte hypertrophy, increased collagen interstitial matrix, uremic autonomic neuropathy, impaired metabolism of potassium, calcium, and phosphate have been suggested as potential mechanisms for QTd prolongation in patients with chronic renal failure. ^5^^, ^^20^^-^^25^ Also, cardiovascular risk factors, which are almost always present in all stages of renal failure, may have a negative impact on cardiovascular physiology. Besides, the presence of cardiovascular risk factors are associated with increased atherosclerotic burden and may lead to some pathological processes, such as medial thickness and calcific lesions resulting in loss of vessel wall compliance.^26^ Depending on these, systolic blood pressure and pulse pressure are increased, left ventricular hypertrophy develops, functional reserve and perfusion of coronary artery are decreased, and myocardial microcirculatory reserve can becompromised. ^26^ QTd prolongation easily measured by surface ECG can be a useful indicator of impaired myocardial microcirculation and repolarization heterogeneity of ventricular myocardium in patients with all stages of renal failure.

***Limitations of the study: ***The small number of patients is the major limitation of this study, and might obscure the definite impact of GFR on QTd. Also, measurement of QTd has some technical limitations. Firstly, the reproducibility of QTd measurement is limited due to intrinsic (T-wave inversion and presence of U-wave, etc.) and extrinsic (interference in the ECG, amplitude, and timing of recording, etc.) factors. ^27^ Secondly, there is not a certain consensus about the normal range of QTd measured by standart 12-lead surface ECG. The normal QTd values reported in literature range from 31±11 ms to 54±27 ms. So, abnormal QTd values distinctly outside of the potential calculation errors (e.g. QTd> 100ms) are suggested as a marker of abnormal ventricular repolarization and heterogeneity. ^27^

In conclusion, this study showed that patients with CAD and poor renal function have increased QTd compared with patients with CAD and good renal function. In order to decrease the frequency of malignant ventricular arrhythmias and sudden cardiac death, QTd should be used as a beneficial, non-invasive,easy reproducible and inexpensive test in the care of patients with poor renal function, especially those with pre-existing cardiac diseases.Further large-scale randomized clinical studies with long follow-up are required toassess more clearly the relationship between eGFR and QTd.

## Authors’ contribution:

*Murat Celik: *study design, manuscript preparation, analysis and interpretation of data, final approval of the version to be published.

*Uyga rCagdas Yuksel:*ECG analysis, data interpretation, literature search, 

*Yalcin Gokoglan:*ECG analysis, data collection, literature search,

*Baris Bugan:*data collection, literature search,

*Emre Yalcin kaya:*data collection, literature search

*Hilmi Umut Unal:*data collection, literature search

*Turgay Celik:*data interpretation, revising the article critically for important intellectual content

*Atila Iyisoy:*data interpretation, revising the article critically for important intellectual content

*Selim Kilic:*statistical analysis, data interpretation

## Notes

***Conflicting of Interest:*** No potential conflicts of interest.

## Notes

***Funding:*** No financial support.

## References

[1] Sarnak MJ, Levey AS, Schoolwerth AC, Coresh J, Culleton B, Hamm LL (2003). Kidney disease as a risk factor for development of cardiovascular disease: a statement from the American Heart Association Councils on Kidney in Cardiovascular Disease, High Blood Pressure Research, Clinical Cardiology, and Epidemiology and Prevention. Circulation.

[2] Brown JH, Hunt LP, Vites NP, Short CD, Gokal R, Mallick NP (1994). Comparative mortality from cardiovascular disease in patients with chronic renal failure. Nephrol Dial Transplant.

[3] Garg AX, Clark WF, Haynes RB, House AA (2002). Moderate renal insufficiency and the risk of cardiovascular mortality: results from the NHANES I. Kidney Int.

[4] Parfrey PS, Foley RN (1999). The clinical epidemiology of cardiac disease in chronic renal failure. J Am SocNephrol.

[5] Lorincz I, Matyus J, Zilahi Z, Kun C, Karanyi Z, Kakuk G (1999). QT dispersion in patients with end-stage renal failure and during hemodialysis. J Am Soc Nephrol.

[6] Wu VC, Lin LY, Wu KD (2005). QT interval dispersion in dialysis patients. Nephrology (Carlton).

[7] Morris ST, Galiatsou E, Stewart GA, Rodger RS, Jardine AG (1999). QT dispersion before and after hemodialysis. J Am SocNephrol.

[8] Jungers P, Massy ZA, Nguyen K, Fumeron C, Labrunie M, Lacour B (1997). Incidence and risk factors of atherosclerotic cardiovascular accidents in predialysis chronic renal failure patients: a prospective study. Nephrol Dial Transplant.

[9] Shulman NB, Ford CE, Hall WD, Blaufox MD, Simon D, Langford HG (1989). Prognostic value of serum creatinine and effect of treatment of hypertension on renal function. Results from the hypertension detection and follow-up program. The Hypertension Detection and Follow-up Program Cooperative Group. Hypertension.

[10] Aras D, Deveci B, Topaloglu S, Çağlı K, Ozeke O, Yıldız A (2006). (Kadınlarda Glomerüler Filtrasyon Hızı Düzeyleri ile Koroner Arter Hastalığının Varlığı ve Şiddeti Arasındaki İlişkinin Değerlendirilmesi) Article in Turkish. Turkiye Klinikleri J Cardiovasc Sci.

[11] Johnson CA, Levey AS, Coresh J, Levin A, Lau J, Eknoyan G (2004). Clinical practice guidelines for chronic kidney disease in adults: Part I. Definition, disease stages, evaluation, treatment, and risk factors. AmFam Physician.

[12] Chobanian AV, Bakris GL, Black HR, Cushman WC, Green LA, Izzo JL (2003). The Seventh Report of the Joint National Committee on Prevention, Detection, Evaluation, and Treatment of High Blood Pressure: the JNC 7 report. JAMA.

[13] Day CP, McComb JM, Campbell RW (1990). QT dispersion: an indication of arrhythmia risk in patients with long QT intervals. Br Heart J.

[14] van de Loo A, Arendts W, Hohnloser SH (1994). Variability of QT dispersion measurements in the surface electrocardiogram in patients with acute myocardial infarction and in normal subjects. Am J Cardiol.

[15] Allan WC, Timothy K, Vincent GM, Palomaki GE, Neveux LM, Haddow JE (2001). Long QT syndrome in children: the value of rate corrected QT interval and DNA analysis as screening tests in the general population. J Med Screen.

[16] Barr CS, Naas A, Freeman M, Lang CC, Struthers AD (1994). QT dispersion and sudden unexpected death in chronic heart failure. Lancet.

[17] Glancy JM, Garratt CJ, Woods KL, de Bono DP (1995). QT dispersion and mortality after myocardial infarction. Lancet.

[18] Beaubien ER, Pylypchuk GB, Akhtar J, Biem HJ (2002). Value of corrected QT interval dispersion in identifying patients initiating dialysis at increased risk of total and cardiovascular mortality. Am J Kidney Dis.

[19] Anastasiou-Nana MI, Nanas JN, Karagounis LA, Tsagalou EP, Alexopoulos GE, Toumanidis S (2000). Relation of dispersion of QRS and QT in patients with advanced congestive heart failure to cardiac and sudden death mortality. Am J Cardiol.

[20] Weber KT, Pick R, Silver MA, Moe GW, Janicki JS, Zucker IH (1990). Fibrillar collagen and remodeling of dilated canine left ventricle. Circulation.

[21] Weber KT, Brilla CG, Janicki JS (1993). Myocardial fibrosis: functional significance and regulatory factors. Cardiovasc Res.

[22] Amann K, Ritz E, Wiest G, Klaus G, Mall G (1994). A role of parathyroid hormone for the activation of cardiac fibroblasts in uremia. J Am Soc Nephrol.

[23] Kirvela M, Yli-Hankala A, Lindgren L (1994). QT dispersion and autonomic function in diabetic and non-diabetic patients with renal failure. Br J Anaesth.

[24] Rombola G, Colussi G, De Ferrari ME, Frontini A, Minetti L (1992). Cardiac arrhythmias and electrolyte changes during haemodialysis. Nephrol Dial Transplant.

[25] Fantuzzi S, Caico S, Amatruda O, Cervini P, Abu-Turky H, Baratelli L (1991). Hemodialysis-associated cardiac arrhythmias: a lower risk with bicarbonate?. Nephron.

[26] Wali RK, Henrich WL (2005). Chronic kidney disease: a risk factor for cardiovascular disease. CardiolClin.

[27] Malik M, Batchvarov VN (2000). Measurement, interpretation and clinical potential of QT dispersion. J Am CollCardiol.

